# Use of e-Cigarettes and Tobacco Products Among Youth in Turkey

**DOI:** 10.5152/eurasianjmed.2022.20168

**Published:** 2022-06-01

**Authors:** Şerif Kurtuluş, Remziye Can

**Affiliations:** 1 Department of Chest Diseases, Harran University Faculty of Medicine, Şanlıurfa, Turkey; 2 Mustafa Kemal Atatürk Vocational and Technical Anatolian High School, Eskişehir, Turkey

**Keywords:** Addiction, e-cigarette, smoking, youth

## Abstract

**Objective:** The aim of this study was to determine high school students’ views about tobacco products and e-cigarettes and e-cigarette smoking prevalence in one of Turkey’s western provinces.

**Materials and Methods:** This cross-sectional study included 286 students. The data collection form consisted of 18 questions related to sociodemographic features and tobacco and e-cigarette use.

**Results:** Of the 286 participants, 32.2% reported having used a tobacco product in the past month, 1.02% reported having used e-cigarettes, and 15.2% have tried e-cigarettes at least once. Of those who tried e-cigarettes, 19.2% of them did so because of curiosity. A positive correlation was found between e-cigarette trial and tobacco use, with age. Smoking/e-cigarette use status was significant with “e-cigarettes are harmful” (*P* = .034), “e-cigarette smoke is harmful” (*P* = .003), and “selling and advertising e-cigarettes is prohibited” (*P* =.043).

**Conclusion:** This study determined that 3 out of 10 youths used tobacco products, and 1 used e-cigarettes. There is a need to raise awareness among high school students and youth regarding the dangers of e-cigarettes and tobacco products.

**Main Points**

- The results of this study show that 3 out of 10 youth in Turkish society use tobacco products, and 1 out of 10 youth try e-cigarettes.
- Although Turkish laws prohibit the use of e-cigarettes, the frequency of use among youth is 3.2%. Young people try e-cigarettes most often because of curiosity.
- This frequency is important for the continuity of nicotine addiction in young people and the risks that may arise.
- Also, our study determined that tobacco use and e-cigarette experience increased with age.

## Introduction

The youth are an important target group for the tobacco industry. According to the 2017 Global Youth Tobacco Survey of Turkey, in total, among young people aged 13-15 years, smoking prevalence is 7.7%, 9.9% in men, and 5.3% in girls.^1^ Other studies involving high school students in Turkey reported the prevalence of smoking between 13.4% and 35.7%.^2-4^ The tobacco industry tries to maintain dependency on the new products it introduces to the market.^5^ One of these products is Electronic Nicotine Delivery Systems (ENDS), known as e-cigarettes. e-Cigarettes cause addiction due to their nicotine content.^5,6^ It is claimed that in causing addiction, e-cigarettes may be a “gateway” in the transition to tobacco smoking. In other words, the previous use of “lighter” substances is a step in the transition to stronger substances.^6,7^ Studies on the long-term health outcomes of e-cigarette use are limited. However, research supports that the e-cigarette is a harmful product.^8^ e-Cigarettes contain many toxic substances, including nicotine,^5^ which have harmful effects on brain development in adolescents and teenagers due to the toxic substances they contain.^5,7^ In fact, studies in the literature suggest that the nicotine solution, heavy metals, glass fibers, and flavoring chemicals in e-cigarettes may have potentially negative effects that may contribute to the pathogenesis of adolescent respiratory tract symptoms and asthma.^9,10^ Studies show that e-cigarette use has become widespread in many European countries, and especially in the United States. In the United States, in 2019, a study reported that 10.5% of secondary school students and 27.5% of high school students had used e-cigarettes in the last 30 days.^11^ A study conducted in Canada reported the frequency of e-cigarette use as 6.5%.^12^ In a study involving 14 352 university students from Belarus, Lithuania, Poland, Russia, and Slovakia, it was reported that 1.1% of the participants used e-cigarettes.^13^ In a study conducted in China, the frequency of e-cigarette use is reported to be 2.1% and 3.6% among vocational high school students.^10^

E-cigarette was started to be sold to the public trough both media and direct marketing in Turkey in 2007.^5^ However, studies on e-cigarettes in Turkey usually cover the adult age group.^14,15^ Studies have shown that the frequency of e-cigarette use among Turkish students is 2.9% and 19% among people over 18 years old.^16,17^ In the literature, no study was found to examine the frequency and use of e-cigarettes among adolescents in Turkey. The aim of this study was to determine the frequency of use of e-cigarettes and other tobacco products and the views on the extent of e-cigarette use among students in a high school in 1 city in western Turkey.

## Materials and Methods

### Study Design

This cross-sectional study was conducted at a vocational school in the city of Eskişehir in the western part of Turkey (n = 286). Eskişehir is an industrial zone where the research is carried out, and its population is 887 475. Eskişehir is considered among liveable cities with its location and socio-economic level.^18^

### Sample

The high school in which the study was conducted consisted of graduate students in the health field. The universe of the research was 724 students enrolled in the 2019-2020 academic year. The sample size calculated for the population of 724 students with a 95% CI and 5% margin of error is 251. Students were informed about the study by the researcher. Written informed consent was obtained from all parents of individual participants. Of these students, 315 provided the signed forms and confirmed that they wanted to participate in the study. Twenty-nine of the forms were incomplete or incorrectly coded in the questionnaire; thus, 286 students completed the study between 1 and December 31, 2019. Of the group, 158 (45.3%) were female. The participants consisted of individuals who reported that they were at most 15 years old (33.21%). Maximum participation was from the 11th-grade level. The majority of respondents reported that their income level was moderate (54.54%). In this study, 92 participants (32.2%) reported that they had used any tobacco product in the last one, and 44 students (15.2%) have tried e-cigarettes at least once. The number of respondents who have been e-smokers within the last 30 days is 3 (3.2%). The product used by the participants consisted mostly of rolled tobacco (34.8%). Of the study group, 194 (67.8%) reported that they bought tobacco product from neighborhood markets. Of the participants, 246 (86.0%) have individuals using tobacco products in their environment (Table 1).

### Ethical Issue

The study was approved by the Harran University Ethical Committee (December 30, 2019; session: 08; decision no: 15).

### Data Collection

The data collection form consisted of 18 questions with 4 questions about sociodemographic characteristics (age, gender, family income status, grade level), 3 questions about tobacco use characteristics and prevalence (Do you know the tobacco products listed below? Do you use tobacco products? Where do you buy tobacco products?), and 11 questions on e-cigarettes categorized as true/false/do not know (e-cigarettes are addictive, e-cigarettes help to quit smoking, e-cigarettes are harmful to health, e-cigarettes contain nicotine, e-cigarettes are at risk of explosion, swallowing the liquid in an e-cigarette cartridge is harmful, e-cigarettes are harmful, e-cigarettes are prohibited in our country, use of e-cigarettes is forbidden in our country, e-cigarettes are produced by tobacco companies), and what are your reasons for trying e-cigarettes?

### Statistical Analysis

Using the IBM 23.0 program (IBM SPSS Corp.; Armonk, NY, USA), sociodemographic characteristics, tobacco use features and prevalence, and the percentage and frequency distributions of the questions related to e-cigarettes are given. Tobacco product use status was categorized as yes/no, and the prevalence of the participants’ tobacco product use was determined. Questions regarding e-cigarette use were compared in terms of statistical significance by chi-square analysis in 2 groups. A Spearman’s correlation analysis was performed between the age variable and the status of tobacco product use and trying e-cigarettes. The reasons for using e-cigarettes are shown in a pie chart.

## Results

Our study determined that a positive relationship existed between the participants’ age variable and tobacco use and trying e-cigarettes (Table 2).

**Figure 1.**
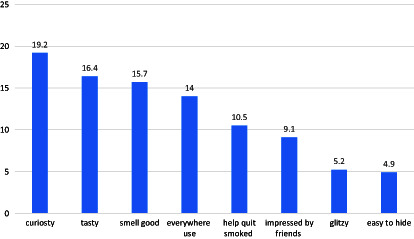
Reasons for participants’ e-cigarette experience.

When the reasons for trying e-cigarettes at least once in their life are listed to participants, the most frequent reason for trying was curiosity (19.2%). In terms of other reasons for trying, 16.4% of the participants reported that the taste of e-cigarettes was good, 15.7% of participants stated they smelled good, 14.0% stated they were available everywhere, and 10.5% were trying to quit smoking (Figure 1).

Of the participants, 251 (87.8%) reported that e-cigarettes are harmful, 239 (83.6%) stated e-cigarettes contain nicotine, 252 (88.1%) reported that e-cigarettes have a risk of explosion, 250 (87.4%) reported that swallowing or inhaling the liquid in the e-cigarette cartridge causes poisoning, and 237 (82.9%) reported that the smoke from the e-cigarette is harmful. There was a statistically significant difference between the participants’ use of any tobacco product and e-cigarettes being harmful (*P* = .034) and the smoke from the e-cigarette being harmful (*P* = .003). Of the participants, 146 (51.0%) reported that e-cigarette advertisement and sales are prohibited in our country. A statistically significant difference was determined between the participants’ tobacco use and their knowledge that e-cigarettes are forbidden to be sold and advertised in our country (*P* = .043). Of the participants, 205 (71.7%) know that using e-cigarettes is prohibited in our country, and 229 (80.1%) of them know that e-cigarettes are produced by cigarette companies. There was no statistically significant difference between the participants’ cigarette/e-cigarette use and the status of knowing that e-cigarette use is prohibited in our country and that e-cigarettes are produced by cigarette companies (Table 3).

## Discussion

In this study, the opinions of Turkish youth about tobacco products, e-cigarette use prevalence, and their views on e-cigarettes were examined. Ninety-two of the participants reported that they use tobacco products, 44 reported they had tried e-cigarettes at least once, and 3 were e-cigarette smokers in the last 30 days (Table 1). It was found that those who tried e-cigarettes did so mostly out of curiosity (Figure 1). The majority of participants (87.8%) think that e-cigarettes are harmful. The frequency of those who know that e-cigarettes contain nicotine is 83.6%. The frequency of those who know that the smoke from e-cigarettes is harmful is 82.9%.

In internal documents, the tobacco industry refers to youth as “replacement smokers” or “learners.”^19^ It is known that around the world, the frequency of tobacco product use, which is increasing especially among youth, will bring addiction in older age and the subsequent burden of diseases that may develop.^20^ This situation, which requires taking significant measures, will have a negative impact on both the national economy and health data. The prevalence of use of any tobacco product in this study was determined to be 32.2%. Our study, which is similar to the literature,^7,13,20^ unfortunately, shows that 3 out of 10 youth are on their way to being addicted.

The e-cigarettes launched by the tobacco industry in 2007 may lead to regular smoking due to both their nicotine content and the structure that mimics cigarette smoking (bringing it to the mouth and holding it).^21^ In our study, it was determined that 44 (15.4%) of 286 students had tried e-cigarettes at least once. This frequency may indicate that at least 1 person will switch to regular smoking. In our study, the number of participants who reported that they were regular e-cigarette users in the last 30 days was 3 (1.2%). In the literature, studies report that the frequency of e-smokers varies between 1.6% and 11.8%.^22-24^ In a study comparing the smoking and e-smoking habits of Spanish and Turkish students in 2019, 2.9% of Turkish students (male) reported that they used e-cigarettes. In the same study, 2.9% of Turkish male students and 13.2% of Turkish female students stated that they only used cigarettes.^16^ The reason for different frequency percentages found in e-cigarette smoking in studies may be the legal barriers to accessing e-cigarettes and information about the dangers of e-cigarettes.

One of the findings we obtained was that the frequency of both tobacco use and e-cigarette experience increased with age (Table 2). Similar to our findings, Vogel et al^22^ reported that at the 16-month follow-up, more than 40% of e-cigarette users (at least once in life) had started smoking. East et al.^23^ in their study conducted in 2018, reported that those in the 16-18 age group found e-cigarettes less harmful than cigarettes compared to individuals in the lower age groups. These results prove that e-cigarettes cause addiction just as other tobacco products do.

It is known that individuals’ lack of information and awareness about a subject causes a feeling of curiosity.^25^ Even if youth do not express that they use tobacco, their interest in these products and the curiosity they may have might be an important public health indicator in terms of future addiction.^26^ Another common reason for using electronic cigarettes reported by both teens and young adults is flavor or taste.^27^ Villanti et al.^28^ with data from the Population Assessment of the Tobacco and Health Survey and the National Tobacco Youth Survey, found that 63-70% of youth tobacco product users choose flavored products.^28^ Although the reasons for trying e-cigarettes vary among youth, they are fundamentally similar. In our study, it was found that youth tried e-cigarettes because of curiosity (19.2%) and the good flavor (16.4%). In addition, it was determined that the smell of e-cigarettes was better than cigarettes (15.7%) and could be used everywhere (although this is false information) (14%) are among the reasons for trying. This finding, which is similar to the literature, shows that youth’s perspective on tobacco products is similar regardless of where they are in the world. Similar to our study findings, in their study conducted with high school and university students who are smokers, Kong et al^29^ found that participants reported that e-cigarettes are a better alternative to cigarettes due to better smell, being able to use e-cigarettes in indoor areas where smoking is prohibited (e.g., movie theatres and schools), and they can hide from parents/teachers because they are odorless. In another study, the most important 3 reasons for using e-cigarettes were curiosity, the influence of friends or family, and quitting smoking.^30^

In our study, it was determined that there was a significant difference between participants’ smoking/e-smoking status and knowing that e-cigarettes are harmful to health. Of the 251 (87.8%) students who stated that e-cigarettes are harmful to health in all study groups, 74 (29.5%) were youth who smoke or use e-cigarettes. Although low according to our study findings, East et al reported that 63.4% of respondents perceived e-cigarettes as less harmful than cigarettes, 22.9% perceived them as equally harmful, and 2.6% as more harmful. While 0.7% in our study stated that they do not know whether e-cigarettes are harmful, this frequency was reported as 11.2% in the study of East et al (2018).^23^ In a study conducted in Egypt, 31.9% of respondents who knew about e-cigarettes believed that e-cigarettes were less harmful than traditional cigarettes.^31^

E-cigarettes contain different amounts of nicotine in their cartridges.^27^ Of the study group, 239 reported that the information that e-cigarettes contain nicotine is correct, and of those, 76 (31.8%) were cigarette/e-smokers. However, there was no difference between knowledge of e-cigarettes containing nicotine and cigarette/e-cigarette use. This result may be related to perception management used in advertising and promoting e-cigarettes. Similarly, in the study of East et al.^23^ no relation was found between the correct perceptions of nicotine harm and e-cigarette use.^23^ Contrary to our study findings, Gorukanti et al^32^ reported that groups differ from each other in terms of addiction among e-cigarettes/cigarettes, both with and without users (e-cigarette/cigarette).^32^

One situation we examined was the risk perception of e-cigarettes. In our study, 252 (88.1%) of the youth reported that e-cigarettes had a risk of explosion, and 250 (87.4%) reported that swallowing or inhaling the liquid in the e-cigarette cartridge caused poisoning. However, there was no difference between the risk of explosion of e-cigarettes and the ingestion or inhalation of liquid in the e-cigarette cartridge causing poisoning and being or not being a cigarette/e-cigarette user. These results can be explained by the insufficient knowledge of e-cigarettes and the lack of addressing this issue among youth. Indeed, studies reporting that youth are e-smokers for social reasons such as curiosity, better smell, ease of use, and being cool rather than mechanical and contextual reasons support our findings.^20^ Despite the studies showing the effects of e-cigarettes on health, studies report that adolescents who previously used cigarettes or e-cigarettes, compared to non-users, stated that e-cigarettes produce only water, do not contain tar, are not addictive, are not tobacco products, produce smoke, feel cleaner, and safer than smoking.^32^

The smoke produced by e-cigarettes is harmful, like other tobacco products. E-cigarettes, like other tobacco products, contain many carcinogenic substances and nicotine and spread from the cartridge to the environment through heating.^33^ In our study, 237 of the participants (82.9%) reported that the smoke from e-cigarettes is harmful. It has been determined that there is a significant difference between the harmful state of the smoke coming out of the e-cigarette and the cigarette/e-cigarette use. This finding is in line with the state of knowing whether smoking/e-cigarettes is harmful or not and use of cigarettes/e-cigarettes, while knowing that swallowing or breathing the fluid in the cartridge of an e-cigarette causes poisoning is in contradiction with the use. However, the results we obtained are promising for youth despite their limited knowledge about e-cigarettes.

Of our participants, 146 (51%)—almost 1 in 2 people, whether they are cigarette/e-cigarette users or not—knew that smoking and advertising e-cigarettes is prohibited in our country. There was a significant difference between smoking cigarettes/e-cigarettes and the sale and advertisement of e-cigarettes being banned in our country. The number of people who knew that use of e-cigarettes is forbidden in our country was 205 (71.7%). However, there was no significant difference between being a cigarette/e-cigarette user or not and the ban on using e-cigarettes in our country. Although the sale and use of e-cigarettes in Turkey is not legal, they can be obtained easily through online e-sales and without any questions. This may indicate that nicotine addiction may continue to gain momentum, especially among youth.

In conclusion, the use of e-cigarettes in the Turkish youth community is seen in only 1 in 100 people, the insufficiency of control mechanisms and legal practices in online systems may increase the prevalence of use. To protect youth, all ENDS should be considered as tobacco products, and the addictive power and negative effects of nicotine in their content should not be ignored. These restrictions should be applied by increasing the restrictions on the sale and advertisement of e-cigarettes. Larger sampling and longitudinal studies are needed to understand the prevalence and risks of using e-cigarettes in Turkish society.

## Limitations

This study had several limitations. The surveys used were self-managed in the school environment. All participants consist of youth in high school. Thus, no generalization can be made for youth in other cities. Secondly, this study is cross-sectional. Therefore, causality cannot be determined. Future research through longitudinal studies should examine how perceptions of harm and benefit are associated with the introduction of e-cigarettes.

## Figures and Tables

**Table 1.** Distribution of Some Features Related to Tobacco Use

	N	%
**Gender**		
Female	158	45.3
Male	128	44.7
**Age**		
14	36	12.58
15	95	33.21
16	91	31.83
17 and above	64	22.37
**Level of education**		
9	33	11.54
10	105	36.71
11	96	33.57
12	52	18.18
**Family income**		
Low	72	25.18
Middle	156	54.54
High	58	20.28
**Do you use any tobacco products?**		
Yes	92	32.2
No	194	67.8
**Have you tried e-cigarettes in your life?**		
Yes	44	15.4
No	242	84.6
**Type of tobacco product used**		
Not used	194	67.8
Cigarette	42	14.7
Wrapping tobacco	32	11.1
Wrapping tobacco + cigarette	15	5.2
E-cigarette	3	1.2
**Presence of smoking in the near environment**		
Yes	246	86.0
No	40	14.0

**Table 2.** Correlation Between Age and Tobacco Use and e-Cigarette Experience

		Tobacco Use	Experience of e-Cigarette
**Age**	**Correlation coefficient**	0.136	0.226
**Sig**	.022	.000

**Table 3.** Opinions According to Tobacco/e-Cigarette Use

	Tobacco/e-Cigarette Use			
	Yes, n (%)	No, n (%)	Total, n (%)	Test Value/P
**e-Cigarettes are harmful**				
True	74 (29.5)	177 (70.5)	251 (87.8)	**6.782/.034**
False	17 (5.5)	16 (48.5)	33 (11.5)	
I don’t know	1 (50.0)	1 (50.0)	2 (0.7)
**e-Cigarettes contain nicotine**				
True	76 (31.8)	163 (68.2)	239 (83.6)	0.073/.787
False	15 (34.1)	29 (65.9)	44 (15.4)	
I don’t know	1 (33.3)	2 (66.7)	3 (1.0)
**e-Cigarette has risk of explosion**				
True	78 (31.0)	174 (69.0)	252 (88.1)	1.309/.253
False	13 (41.9)	18 (58.1)	3 (10.8)	
I don’t know	1 (33.3)	2 (66.7)	3 (1.0)
**Swallowing or inhaling the liquid in the e-cigarette cartridge caused poisoning**				
True	77 (30.8)	173 (69.2)	250 (87.4)	2.752/.097
False	13 (46.4)	15 (53.6)	28 (9.8)	
I don’t know	2 (25.0)	6 (75.0)	8 (2.8)
**Smoke from e-cigarettes is harmful**				
True	67 (28.3)	170 (71.7)	237 (82.9)	**8.550/.003**
False	23 (53.5)	20 (46.5)	43 (15.0)	
I don’t know	2 (33.3)	4 (66.7)	6 (2.1)
**E-cigarette is forbidden to sell and advertise in our country**				
True	39 (26.7)	107 (73.3)	146 (51.0)	**4.089/.043**
False	50 (38.8)	79 (61.2)	129 (45.1)	
I don’t know	3 (27.3)	8 (72.7)	11 (3.8)
**Use of e-cigarette is prohibited in our country**				
True	61 (29.8)	144 (70.2)	205 (71.7)	2.275/.312
False	27 (39.7)	41 (60.3)	68 (23.8)	
I don’t know	4 (30.8)	9 (69.2)	13 (4.5)
**E-cigarette is produced by cigarette companies**				
True	72 (56.8)	157 (68.6)	229 (80.1)	3.492/.174
False	16 (43.2)	21 (56.8)	37 (12.9)	
I don’t know	4 (20.0)	16 (80.0)	20 (7.0)
